# Zhilong Huoxue Tongyu Capsules Ameliorate Early Brain Inflammatory Injury Induced by Intracerebral Hemorrhage *via* Inhibition of Canonical NFкβ Signalling Pathway

**DOI:** 10.3389/fphar.2022.850060

**Published:** 2022-03-31

**Authors:** Maryam Mazhar, Guoqiang Yang, Linshen Mao, Pan Liang, Ruizhi Tan, Li Wang, Houping Xu, Luyin Yang, Wei Ren, Sijin Yang

**Affiliations:** ^1^ National Traditional Chinese Medicine Clinical Research Base and Drug Research Center, The Affiliated Traditional Chinese Medicine Hospital of Southwest Medical University, Luzhou, China; ^2^ Institute of Integrated Chinese and Western Medicine, Southwest Medical University, Luzhou, China; ^3^ Research Center for Integrated Chinese and Western Medicine, The Affiliated Traditional Chinese Medicine Hospital of Southwest Medical University, Luzhou, China; ^4^ Research Unit of Molecular Imaging Probes, Department of Radiologic Technology, Faculty of Associated Medical Sciences, Chiang Mai University, Chiang Mai, Thailand; ^5^ Preventive Treatment Center, The Affiliated Traditional Chinese Medicine Hospital of Southwest Medical University, Luzhou, China

**Keywords:** intracerebral hemorrhage, traditional Chinese medicine, TNFα-NFкB signalling, inflammatory cytokines, inflammatory brain injury

## Abstract

**Background:** Intracerebral hemorrhage (ICH) is a debilitating and fatal condition with continuously rising incidence globally, without effective treatment available. *Zhilong Huoxue Tongyu* (ZLHXTY) capsule is a traditional Chinese medicine that is used for ICH treatment in China. However, the evidence based mechanism is not clear.

**Purpose:** To study the protective effects of ZLHXTY capsules against ICH pathogenesis *via* targetting nuclear factor kappa *β* (NFкβ) canonical signalling pathway.

**Methods:** C57BL/6 J mice ICH models using autologous blood injection were used to study the effect of ZLHXTY (1.4 g/kg P.O.) after 24 and 72 hrs of ICH induction. The neurological scoring, corner turn test and balance beam with scoring was performed to assess neurological damage. Hematoxylin/eosin and nissl staining was used for histopathological evaluation. Levels of TNFα, NFкB, iNOS, COX2, IL1, IL6 were measured using real time qPCR and western blotting. Protein levels of IKKβ and IкBα were analyzed through western blotting. Immunofluorescence for co-expression of NeuN/TNFα, NeuN/NFкB, Iba1/TNFα, and Iba1/NFкB was also performed.

**Results:** Treatment with ZLHXTY capsules after ICH ameliorated inflammatory brain injury after 24 and 72 h; revealed by neurological scoring, hematoxylin/eosin and nissl staining. The qPCR and western blot analyses demonstrated significant downregulation of TNFα, NFкB, iNOS, COX2, IL1β and IL6. Further, the IKKβ and IкBα revealed significant downregulation and upregulation respectively in western blot. Immunofluorescence also revealed attenuated expression of TNFα and NFкB in neurons and also low expression of Iba1.

**Conclusion:** ZLHXTY capsules elicit its neuroprotective effect by targetting the NFкβ canonical signalling pathway, thereby ameliorating the ICH induced brain injury.

## Highlights

• NFкB signalling has major implication in ICH induced brain injury.
• ZLHXTY capsule provide neuroprotection at earlier stages of ICH *via* inhibiting NFкB signalling pathway.
• ZLHXTY capsule can serve as better treatment option for ICH, primarily through anti-inflammatory effect.

## Introduction

Intracerebral hemorrhage (ICH) is a fatal and devastating cerebrovascular disease that accounts for 15% of all strokes. ICH is associated with high rate of mortality and morbidity (Krishnamurthi et al., 2014). The global incidence of ICH continues to rise, affecting  5 million people each year worldwide, including  3 million deaths and only 12–39% of survivors resuming functional independence (van Asch et al., 2010; Krishnamurthi et al., 2014; An et al., 2017). ICH is characterized by bleeding within brain parenchyma due to rupture of blood vessels causing mass effect and cerebral damage (Qureshi et al., 2009). Hypertension is the main cause of ICH (Qureshi et al., 2001). The mechanism of ICH is complex. The primary injury begins with the onset of bleeding and activation of inflammatory mechanisms which progressively leads to secondary brain injury that reach its peak in 3–7 days (Qureshi et al., 2001; Qureshi et al., 2009). Besides the inflammatory role of activated immune cells i-e., neutrophils, monocytes, astrocytes and dendritic cells; the blood derived components such as heme, iron and thrombin aggravate the ICH induced brain injury (Aronowski and Zhao, 2011). Accumulating evidence suggests that blood derived free radicals, cytokines and glutamate receptors, lead to the activation of nuclear factor kappa B (NFκB) (Wagner, 2007) within minutes after the onset of ICH that lasts for a week (Zhao et al., 2007). NFкB is a master regulator of inflammation and anticipated to cause neuronal apoptosis in perihematomal regions after ICH (Hickenbottom et al., 1999; Wang et al., 2011a).

Despite improvement in current knowledge of ICH, there is still lack of effective treatments, development of which is urgently required (Liddle et al., 2020). Traditional Chinese medicine (TCM) is the prominent medical specialty from pre-historic era in China (Zhao et al., 2021). Recently, TCM has also been recognized by World Health Organization (WHO) and is adopted in the eleventh revision of the International Statistical Classifcation of Diseases and Related Health Problems (ICD-11) (Lam et al., 2019). WHO encourages the provision of traditional and complementary medicines in the mainstream medical and healthcare services that contribute to achieve the Sustainable Development Goal 3 (SDG 3) of universal health coverage (UHC) (World Health Organization, 2019).

Zhilong Huoxue Tongyu (ZLHXTY) capsule is an approved (Patent No. 200810147774.1) hospital preparation of Affiliated Traditional Chinese Medicine Hospital, Southwest Medical University, Luzhou China, designed by Professor S.J. Yang according to the Buyang Huanwu decoction method based on Xuan Fu theory (Liu et al., 2011; Wu et al., 2015). It consists of a mixture of five herbs (Table 1), each having its own benefit according to Chinese theory of medicines. *Hirudo nipponica* Whitman and *Pheretima Aspergillum* (E. Perrier) is widely known for thrombolytic properties, removal of blood stasis syndrome and for the treatment of cerebral and cardiovascular diseases (Dong et al., 2016; Cheng et al., 2020). *Astragalus membranaceus* Fisch. ex Bunge or *Astragalus mongholicus* Bunge nourishes “Qi” and used for the treatment of “Qi deficiency” syndrome (Qi et al., 2017). *Cinnamomum cassia* (L.) J. Presl or *Neolitsea cassia* (L.) Kosterm., is cardioprotective, neuroprotective, immunoregulatory, analgesic and anti-inflammatory (Zhang et al., 2019). *Sargentodoxa cuneata* (Oliv.) Rehder and E.H. Wilson or *Holboellia cuneata* Oliv. is also known for dissipating blood stasis, pain relief and anti-inflammatory effects (Bai et al., 2019). Intriguingly, all these herbs show anti-inflammatory effect through inhibition of NFкB signalling mechanism (Dong et al., 2016; Qi et al., 2017; Bai et al., 2019; Zhang et al., 2019; Cheng et al., 2020).

**TABLE 1:** Components of Zhilong Huoxue tongyu capsule.

Scientific Name	Family	English Name	Chinese Name	Part Used	Quantity (Dry Weight)
*Hirudo nipponica* Whitman	Hirudideae	Leech	ShuiZhi	Dried whole animal	0.32 g
*Pheretima aspergillum* (E. Perrier)	Megascolecidae	Earthworm	Guang Dilong	Dried whole animal	1.7 g
*Astragalus membranaceus* Fisch. ex Bunge or *Astragalus* mongholicus Bunge	Fabaceae	*Astragalus*	Huang Qi	Roots	2.3 g
*Cinnamomum cassia* (L.) J.Presl or Neolitsea cassia (L.) Kosterm	Lauraceae	Cassia	GuiZhi	Stem/Twig	0.86 g
Sargentodoxa cuneata (Oliv.) Rehder and E.H.Wilson or *Holboellia cuneata* Oliv	Lardizabalaceae	Sargentgloryvine	Da XueTeng	Stem/Twig	1.7 g

Although, ZLHXTY capsules have been used clinically for treating various cardiovascular and cerebrovascular diseases for about 20 years, but the details of evidence based mechanism of action on ICH is lacking (Liang et al., 2021). Therefore, in the current study, we aim to identify the anti-inflammatory effect of ZLHXTY capsules in early stages of ICH induced brain injury *via* targetting NFкβ signalling pathway.

## Materials and Methods

### Materials

Clinically used ZLHXTY capsules were obtained from the pharmacy department of the Affiliated Traditional Chinese Medicine Hospital of Southwest Medical University, Luzhou, Sichuan, China. Standard compounds for HPLC quality control analysis including L-epicatechin, calycosin-7-O-β-glucoside, coumarin, ononin, calycosin, cinnamaldehyde, formononetin, and wogonin were purchased from Beijing Saibaicao Technology Co., Ltd. (Beijing, China). All the solvents such as ethanol, acetonitrile, formic acid and ammonium formate were of HPLC-grade and were purchased from Thermo Fisher Scientific (Massachusetts, USA). Hematoxylin & Eosin staining kit was purchased from Beyotime biotechnology, Shanghai, China (Cat. No. C01015-1). Nissl staining kit was purchased from Solarbio, Beijing, China (Cat. No. G1430). The list of primary antibodies used in this study are given in Table 2. Fluorescently labeled secondary antibodies for western blot i.e., goat anti-mouse IgG Alexa Fluor 790 (A11357), goat anti-rabbit IgG Alexa Fluor 680 (A21109); and for immunofluorescence i.e., goat anti-rabbit IgG Alexa Fluor 555 (A21429), goat anti-mouse IgG Alexa Fluor 488 (A11001) were purchased from Invitrogen Life technologies, United States.

**TABLE 2:** List of primary antibodies used.

Antibody	Type	Dilution	Uniprot RRIDs	Catalogue number	Source
TNFα	Monoclonal	1:1000	P01375	sc-52746	Santa Cruz Biotechnology, Inc., CA,United States.
NFκB-p65	Monoclonal	1:1000	Q04206	sc-8008	
COX2	Monoclonal	1:1000	P35354	sc-166475	
IKKβ	Monoclonal	1:1000	O14920	sc-8014	
IкBα	Monoclonal	1:1000	P25963	sc-1643	
IL6	Monoclonal	1:1000	P05231	sc-32296	
IL1β	Polyclonal	1:1000	P01584	D320820	Sangon Biotech Co., Ltd. Shanghai, China
NFKBIA (Phospho-Ser32/Ser36)	Polyclonal	1:1000	P25963	D155066	
iNOS	Monoclonal	1:1000	P29477	13120	Cell Signalling Technologies Inc., Shanghai, China
NeuN	Monoclonal	1:1000	A6NFN3	D4G4O	
Iba1/AIF-1	Monoclonal	1:1000	P55008	E404W	
NFκB-p65 (Phospho-Ser536) (93H1)	Monoclonal	1:1000	Q04206	3033S	
IKKβ(PhosphoY199)	Polyclonal	1:1000	O14920	ab59195	Abcam
GAPDH	Monoclonal	1:10000	P04406	AB0037	Abways Technology, Inc., Shanghai, China

### Sample Preparation for UPLC-HRMS Analysis

For the ultra-high performance liquid chromatography coupled high resolution mass spectrometry (UPLC-HRMS) based chemical characterization of ZLHXTY capsule, firstly, the dried whole bodies of *Hirudo nipponica* Whitman and twigs of *Cinnamomum cassia* (L.) J. Presl (or Neolitsea cassia (L.) Kosterm.) were grinded together into a fine powder. The rest of the herbs, *Pheretima aspergillum* (E. Perrier), *Astragalus membranaceus* Fisch. ex Bunge (or *Astragalus* mongholicus Bunge), Sargentodoxa cuneata (Oliv.) Rehder and E.H.Wilson (or *Holboellia cuneata* Oliv.), were soaked in water to make decoction concentrate. Followed by drying, the herbs were grinded to make into the fine powder. The two powders were mixed together to prepare ZLHXTY mixture. Later, the ZLHXTY powder was extracted in ethanol (1:8 volume ratio) under sonication for 30 min to obtain the ethanolic extract or supernatant A. Then, the water was added in the filter residue (1:10 volume ratio) under sonication for 30 min to obtain the water extract or supernatant B. Finally, the two supernatants A and B were combined (1:1 volume ratio) together, concentrated by rotary evaporation and freeze vacuum drying to obtain extracts of ZLHXTY capsule for subsequent UPLC-HR-MS analysis.

The eight standard compounds L-epicatechin, calycosin-7-O-β-glucoside, coumarin, ononin, calycosin, cinnamaldehyde, formononetin, and wogonin, were firstly extracted with ethanol followed by high speed centrifugation to collect the supernatant that was later used for standard validation of ZLHXTY capsule under UPLC-HRMS technique.

### Conditions Optimization for UPLC-HRMS

The chemical characterization analysis of the ZLHXTY capsule extract was operated on Ultimate 3000 hyperbaric LC system coupled with high resolution Q-Exactive mass spectrometer *via* an electrospray ionization (ESI) interface (Thermo Fisher Scientific, Bremen, Germany), using BEH C18 column (1.7 μm, 2.1 mm ID × 100 mm, Waters) maintained at 35°C. Following are the optimized chromatographic parameters of our study: mobile phase was composed of water (0.1% formic acid, A) mixed in gradient mode with acetonitrile (0.1% formic acid, B), at a flow rate of 200 μL/min. The elution gradient was optimized as follows: 0–5 min, 2% B; 5–8 min, 2–20% B; 8–45 min, 20–55% B; 45–52 min, 55–100% B; 52–58 min, 100% B. The injection volume was 2.0 μL and the sampler was set at 4°C.

Positive full scan modes within the range of m/z (mass/charge ratio) 150-1500 at a resolution of 70,000 were used for acquisition of accurate molecular ion. The other parameters were set as follows: spray voltage, +3.5 kV; sheath gas flow rate, 35 arb; aux gas flow rate, 10 arb; capillary temperature, 320°C; vaporizer temperature, 250°C; RF lens, 50%. Xcalibur 3.0 software (Thermo Fisher) was used for UPLC-HRMS control and data handling.

### Animals

The male C57BL/6 J mice (20–22 g body weight, 7–8 weeks old) were raised in regular and clean cages under maintained conditions of temperature at 22 ± 0.5°C, humidity 55 ± 5%, with 12-h alternate light-dark cycles. All animals were allowed free access to standard animal chow and water. The study was performed according to the National Institute of Health (NIH) Guide for the Care and Use of Laboratory Animals and approved by the Animal Research Committee of Southwest Medical University, Luzhou, China.

### Intracerebral Hemorrhage Model

ICH was induced by infusion of autologous blood. Mice were anesthetized with an intraperitoneal injection of sodium pentobarbital at a dose of 50 mg/kg. Anesthetized mice were placed in stereotaxic frame with the head being carefully and firmly fixed in the apparatus. A small cut is made in the skin of the head followed by application of 30% H_2_O_2_ on the surface of skull to remove the periosteum and clarify the skull joints. A 1-mm burr hole was drilled at co-ordinates of 2 mm lateral and 0.2 mm anterior to the bregma in the right striatum of mice brain. A volume of 25 μL of autologous blood was collected in the Hamilton syringe from a tail cut and then injected in the brain through that burr hole with a needle insertion depth of 3 mm. Blood infusion flow was maintained at a rate of 5 μL/min. After completion of blood infusion, the needle was kept in place for 5 min to prevent backflow of blood, and after then the needle was withdrawn slowly and carefully. Then skin was sutured in aseptic conditions and the animals were allowed to recover and regain consciousness in a warm environment maintained at 37°C.

### Treatment Groups

The animals were randomly divided into five groups (*n* = 18); 1) normal control, 2) ICH model group; and treatment groups 3) Low dose ZLHXTY-LD (0.35 g/kg) 4) Medium dose ZLHXTY-MD (0.7 g/kg), and 5) High dose ZLHXTY-HD (1.4 g/kg). The first ZLHXTY dose was administered orally within 2 h of ICH induction after mice regain consciousness, and continued as once daily dosing for 3 days. The mice in normal control group and ICH group received the same volume of normal saline orally. At 24 and 72 hrs the mice were killed to assess the neuroprotective effect of ZLHXTY at different time points. We observed the dose dependent effect of ZLHXTY capsules treatment in our experiments. The data for neurological scoring represent the effect of all three doses of ZLHXTY capsules treatment. For the subsequent experiments, the data presented here used the highest effective dose of ZLHXTY-HD 1.4 g/kg.

### Behavioral Tests

Behavioral tests were carried out 2 days before and after 6, 24, 48, and 72 h of ICH induction and the average score was obtained by observations form two independent observers, blinded to the experimental design. The tests included 28 point neurological scoring test (Clark et al., 1997), balance beam test (Feeney et al., 1982; Liu et al., 2020) and corner turn test (Krafft et al., 2014).

#### Neurological Score:

Twenty-eight point neurological scoring was employed (Table 3). The higher the score, the severe the injury (Clark et al., 1997).

**TABLE 3:** Focal deficits (0-28) scoring scale (Clark et al., 1997).

Score	0	1	2	3	4
Body Symmetry (open bench top)	Normal	Slight asymmetry	Moderate asymmetry	Prominent asymmetry	Extreme asymmetry
Gait (open bench top)	Normal	Stiff, inflexible	Limping	Trembling, drifting, falling	Does not walk
Climbing (gripping surface, 45° angle)	Normal	Climbs with strain, limb weakness present	Holds onto slope, does not slip or climb	Slides down slope, unsuccessful effort to prevent fall	Slides immediately, no effort to prevent fall
Circling behavior (open bench top)	Not present	Predominantly one-sided turns	Circles to one side (not constantly)	Circles constantly to one side	Pivoting, swaying, or no movement
Front limb symmetry (mouse suspended by its tail)	Normal	Light asymmetry	Marked asymmetry	Prominent asymmetry	Slight asymmetry, no body/limb movement
Compulsory circling (front limbs on bench, rear suspended by tail)	Not present	Tendency to turn to one side	Circles to one side	Pivots to one side sluggishly	Does not advance
Whisker response (light touch from behind)	Symmetrical response	Light asymmetry	Prominent asymmetry	Absent response ipsilaterally, diminished	Absent proprioceptive response bilaterally
				contralaterally	

#### Balance Beam Test

The mice were placed on a beam (2 cm), and the latency period to reach the home cage was recorded. The maximum time limit for observation was 60 seconds (Liu et al., 2020). The number of paw slips were also recorded and overall behaviour of animal while on beam was measured according to the scoring criteria. The higher the score, the more serious the neurological damage (Table 4) (Feeney et al., 1982). All the mice were trained on the balance beam apparatus for 2 days prior to induction of ICH.

**TABLE 4:** Neurological scoring system from beam walking^a^.

Score	Performance on the beam
7	Traverses beam normally with both affected paws on horizontal beam surface, neither paw ever grasps the side surface, and there are no more than two footslips; toe placement style is the same as preinjury
6	Traverses beam successfully and uses affected limbs to aid >50% of steps along beam
5	Traverses beam successfully but uses affected limbs in <50% of steps along beam
4	Traverses beam and, at least once, places affected limbs on horizontal beam surface
3	Traverses beam by dragging affected hindlimbs
2	Unable to traverse beam but places affected limbs on horizontal beam surface and maintains balance for ≥5 s
1	Unable to traverse beam; cannot place affected limbs on horizontal beam surface

a Adapted from the method of Feeney et al. (25) used to evaluate unilateral lesions of sensory cortex in rats.

#### Corner Turn Test:

Mice were allowed to proceed into a 30 degrees corner and the direction in which mice turn either left or right by placing one or both forelimbs on the wall as it shifts it weight around was observed. The number of right turns within 60 seconds were recorded as percentage (Krafft et al., 2014).

### Animal Surgery and Specimen Preparation

After completion of respective dosing regimen, the mice were terminally anesthetized with overdose of sodium pentobarbital then transcardially perfused with phosphate buffered saline followed by 4% paraformaldehyde. Later, the mice were decapitated and brains were collected and processed accordingly for histology and immunofluorescence studies. For qPCR and western blot experiments, the transcardial perfusion with 4% paraformaldehyde was omitted and the brain tissues were collected in RNase free eppendorfs and snap-frozen in liquid nitrogen and stored in −80°C for later use.

### Hematoxylin and Eosin and Nissl Staining

As described earlier, the perfusion fixed brain tissues were further underwent immersion fixation in 4% formaldehyde overnight. Then, the tissues were dehydrated in a series of graded alcohols 50, 60, 70, 80, 90, 100% and then in xylene for 30 min each. Later, the brain tissues were paraffin embedded and paraffin sections of 4 µm thickness at coronal plane were cut using microtome (Leica RM 2245, Wetzlar, Germany). For staining, the tissue slides were rehydrated using xylene, 100% alcohol, 90, 80, 70, 60, 50% alcohol, and then water. HE staining was carried out according to the standard procedure. Similarly, nissl staining was performed according to manufacturer’s instructions. The slides were observed under Leica DM500 microscope equipped with Leica ICC50W camera and images were captured using software Leica application suite X, at magnification 400 x .

### Quantitative Real Time Polymerase Chain Reaction

The qRT-PCR was used to analyse the mRNA levels of NFкβ-P50, TNFα, IL1β, IL6, COX2 and iNOS at 24 and 72 h after ICH (*n* = 9 per group per time-point). Total RNA was extracted from hemorrhagic cortex using the Trizol reagent (Beyotime Biotechnology, China, Cat# R0016); reverse-transcribed into cDNA using HiScript III RT SuperMix for qPCR (+gDNA wiper) kit (Vazyme Biotech Co.,Ltd., China, Cat. No.R323-01) and qRT-PCR was performed on LightCycler^®^ 480 Instrument II (Roche, USA) in the presence of a fluorescent dye ChamQ Universal SYBR qPCR Master Mix (Vazyme Biotech Co.,Ltd., China, Cat. No.Q711-02/03). Absorbance was read at 260 and 280 nm using an UV spectrophotometer and the RNA samples with an OD260/OD280 value >1.8 were only considered appropriate for use. The mRNA level was normalized to the GAPDH and was calculated by the 2^−ΔΔCt^ method. The primer sequences are given in table 5.

**TABLE 5:** Sequences of Primers (5′- 3′) for qPCR.

Gene Name	Primer sequence	Product Length
NFкβ-P50	F: GGA​GGC​ATG​TTC​GGT​AGT​GG	20
	R: CCC​TGC​GTT​GGA​TTT​CGT​G	19
TNFα	F: CAT​CTT​CTC​AAA​ATT​CGA​GTG​ACA​A	25
	R: TGG​GAG​TAG​ACA​AGG​TAC​AAC​CC	23
IL1β	F: TGC​CAC​CTT​TTG​ACA​GTG​ATG	21
	R: AAG​GTC​CAC​GGG​AAA​GAC​AC	20
IL6	F: AAA​GAG​TTG​TGC​AAT​GGC​AAT​TCT	24
	R: AAG​TGC​ATC​ATC​GTT​GTT​CAT​ACA	24
COX2	F: TGA​GCA​ACT​ATT​CCA​AAC​CAG​C	22
	R: GCA​CGT​AGT​CTT​CGA​TCA​CTA​TC	23
iNOS	F: TTG​GAG​CGA​GTT​GTG​GAT​TG	20
	R: GGTCGTAAT GTCCAG GAAGTAGG	23
GAPDH	F: CGG​AGT​CAA​CGG​ATT​TGG​TCG​TAT	24
	R: AGC​CTT​CTC​CAT​GGT​GGT​GAA​GAC	24

### Western Blot Analysis

Brain samples were homogenized with RIPA buffer containing 1 mM PMSF on ice. After centrifugation, the supernatants were collected and protein concentrations were measured using Bradford Coomassie brilliant blue method. From each sample, 40 µg protein was separated by 12% SDS-PAGE and transferred to nitrocellulose membranes. After blocking with 5% skimmed milk for 1 h, the membranes were incubated with various primary antibodies mentioned in table 2, overnight at 4°C. Next day, the blots were washed with TBST 5 min x3 and then incubated with near infrared fluorescently labeled secondary antibodies at concentration 1:10,000, at room temperature for 1 h. Then, membranes were washed with TBST 5 min x3. Fluorescent detection was performed using the Amersham Typhoon™ laser scanner (Cytiva, USA). Protein levels were normalized with respect to GAPDH and quantified using the ImageJ software (NIH, Bethesda, MA, USA).

### Double Immunofluorescence Staining

Following transcardial perfusion with PBS and 4% paraformaldehyde, the brain tissues were carefully removed out and kept in 30% sucrose solution overnight a 4°C. After the tissues were sank down in the sucrose solution, the brains were cryopreserved in Tissue-Tek^®^ O.C.T.medium (Sakura, Japan) and snap frozen in liquid nitrogen. Cryosections of 7 µm thickness at coronal plane were cut using a freezing microtome (Leica CM 1950; Wetzlar, Germany) and taken onto slides followed by 20 min perfusion with 0.3% Triton X-100 at room temperature. Later, the sections were washed with PBS 5 min x3 with subsequent blocking in 5% BSA for 1 h at room temperature. After blocking, the sections were incubated in a mixture of primary antibodies i.e., NeuN + TNFα, NeuN + NFкB, Iba1+TNFα and Iba1+NFкB, at a dilution of 1:1000 at 4°C overnight. Next day, the sections were washed with PBS 5 min x3 and then incubated in respective secondary antibody dilutions (1:1000) in dark for 1 h at room temperature. Next, the sections were incubated with DAPI for 10 min followed by washing in PBS 5 min x3 and coverslip mounting in glycerol. The slides were kept in dark and observed under Leica DM4 B fluorescence microcope equipped with Leica DMC6200 camera. The images were captured using Leica application suite X software at magnification 400x.

### Statistical Analysis

The data analysis was performed using two-way analysis of variance (ANOVA), followed by Tukey’s post-hoc tests using GraphPad Prism Version 8.0.1 software (GraphPad Software Inc., CA, USA). A value of *p* < 0.05 was considered statistically significant. Data are shown as the mean ± SD. All the experiments were repeated at least three independent times.

## Results

### Qualitaive UPLC-HRMS Analysis of ZLHXTY Capsules

**FIGURE 1:**
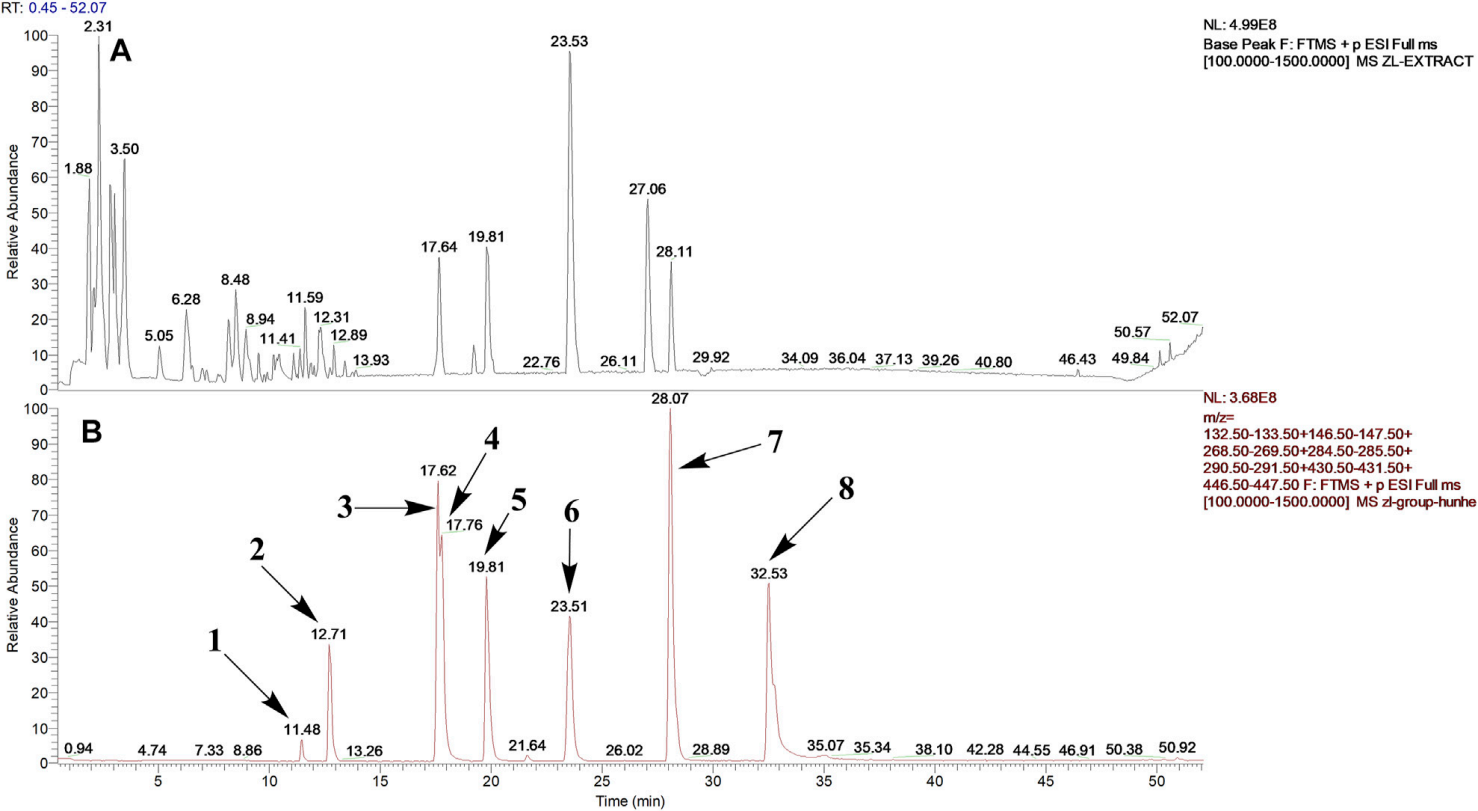
Representative UPLC-HRMS chromatogram of ZLHXTY capsule. **(A)** Chromatogram of ZLHXTY capsule in positive ion modes within the range of m/z 150-1500 at a resolution of 70,000; **(B)** chromatogram of mixed chemical standards, L-epicatechin, calycosin-7-O-β-glucoside, coumarin, ononin, calycosin, cinnamaldehyde, formononetin, and wogonin.

After optimization of chromatographic conditions, the UPLC-HRMS analysis for quality control of ZLHXTY capsules was conducted to obtain the chromatogram (Figure 1A). Eight standard compounds were confirmed in ZLHXTY capsules, including L-epicatechin, calycosin-7-O-β-glucoside, coumarin, ononin, calycosin, cinnamaldehyde, formononetin, and wogonin (Figure 1B). The analytical details of those identified compounds are summarized in table 6.

**TABLE 6:** Representative compounds identified in UPLC-HR-MS analysis of ZLHXTY capsules.

ZLHXTY capsule components	Active Ingredients	Peak no	Retention time (min)	Ion mode	Formula	Molecular weight	Class	ppm	Structure
*Astragalus membranaceus* Fisch. ex Bunge or *Astragalus* mongholicus Bunge	Calycosin-7O-β-D-glucoside	2	12.71	[M + H]^+^	C22H23O10	447.1272	Flavone	−3.07	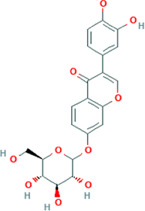
	Ononin	4	17.76	[M + H]^+^	C_22_H_23_O_9_	431.1324	Flavone	−3.04	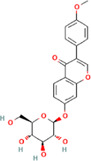
	Calycosin	5	19.81	[M + H]^+^	C16H13O5	285.0746	Flavone	−3.96	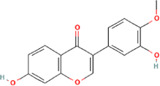
	Formononetin	7	28.11	[M + H]^+^	C_16_H_13_O_4_	269.0796	Flavone	−4.74	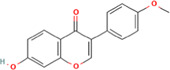
	Wogonin	8	32.53	[M + H]^+^	C_16_H_13_O_5_	285.0745	Flavone	−4.32	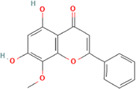
*Cinnamomum cassi*a (L.) J.Presl or Neolitsea cassia (L.) Kosterm	Coumarin	3	17.64	[M + H]^+^	C_9_H_7_O_2_	147.0436	Cinnamic acid	−3.24	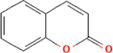
	Cinnamaldehyde	6	23.51	[M + H]^+^	C_9_H_9_O	133.0644	Cinnamaldehyde	−2.79	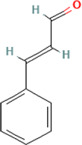
Sargentodoxa cuneata (Oliv.) Rehder and E.H.Wilson or *Holboellia cuneata* Oliv	L-Epicatechin	1	11.48	[M + H]^+^	C15H15O6	291.0853	Flavanol	−3.38	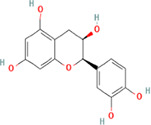

### ZLHXTY Capsules Improve the Neurological Outcome After ICH

**FIGURE 2:**
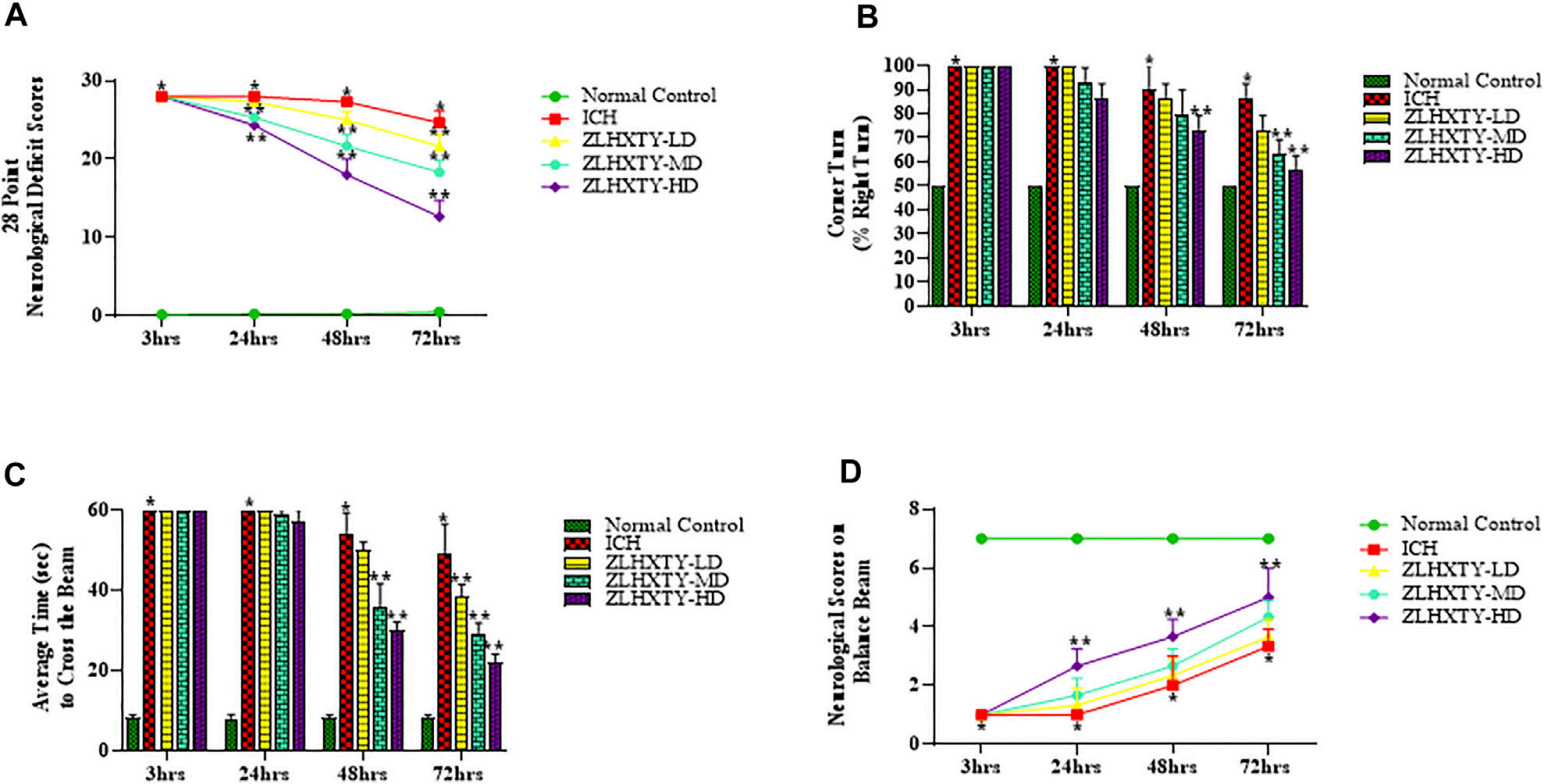
Effect of ZLHXTY capsules on neurological outcomes in mice after ICH. **(A)** 28 point neurological deficit score, **(B)** corner turn test (% right turn), **(C)** balance beam test and **(D)** neurological scoring on balance beam; revealed significant alleviation of neurological deficits after ZLHXTY capsules treatment after 72 hrs of ICH. Data represent the mean ± SD, *n* = 18, *p < 0.05 as compared to normal control, **p < 0.05 as compared to ICH group.

To investigate the neuroprotective role of ZLHXTY after ICH, we determined the neurological deficit score, corner turn test and balance beam test with neurological scoring, starting from 3 hrs of ICH induction followed by 24, 48 and 72 h observation time points. Modified 28 point neurological deficit scores are shown in Figure 2A. All the mice in normal control group were healthy with no obvious neuromotor dysfunction, while ICH group had demonstrated severe and significant (*p < 0.05) neurological impairment at all time points after ICH induction. However, ZLHXTY treated group had shown gradual improvement and significant (**p < 0.05) recovery.

We also determined the occurrence and severity of ICH damage by corner turn test. Since we induced the ICH in the right basal ganglia, motor and sensory functions of the left side of the body were paralyzed and affected, causing significant (*p < 0.05) increase in the frequency of right turns in ICH group (almost 100%) as compared with the normal group. However, the ZLHXTY treatment groups demonstrated gradual, progressive and significant recovery (**p < 0.05) than ICH group. The baseline of the corner turn test result from normal group was about 50%, as the probability of left or right turn was basically equal (Figure 2B).

Further, we also performed the balance beam test using a rating scale to measure the motor co-ordination and balance. The mice in ICH group showed increased fear and inability to move after 3 and 24 h. However, after 48 and 72 hrs, the animals in ICH group demonstrated slight ability to walk on the beam with severe difficulty and showed frequent paw slips as compared to the normal control (Figure 2C). The ZLHXTY treated mice showed increased fear, frequent paw slips and severe difficulty in crossing the beam until 24 h, however, the performance was improved significantly (**p < 0.05) after 48 hrs and further progressed after 72 h (Figure 1C). The balance beam motor deficit score is shown in Figure 2D.

### ZLHXTY Capsules Protect the Brain Parenchyma and Neurons Post-ICH

**FIGURE 3:**
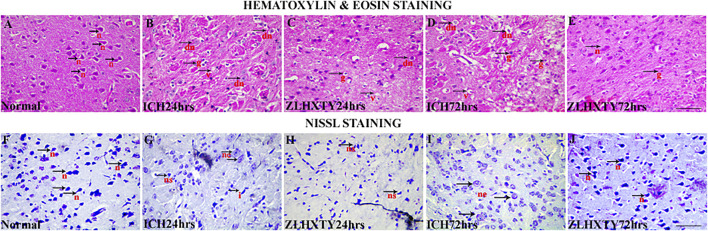
HE and Nissl staining of brain showing neuroprotective effect of ZLHXTY treatment after ICH. HE staining **(A–E)**, showing **(A)** normal, black arrows with n-normal pyramidal neurons and intact parenchyma; **(B,D)** 24 and 72 h ICH pathological changes, black arrows with v-vacuolation and parenchymal loss, dn-neuronal shrinkage and degeneration, g-reactive gliosis; **(C,E)**—24 and 72 h ZLHXTY-HD treatment, improving inflammatory histopathological changes with more intact neuropil and less vacuolation, less neuronal damage, less number of glial cells and inflammation. Nissl staining **(F–J)**, **(F)** normal, black arrows indicate normal nissl bodies; **(G,I)** 24 and 72 h ICH injury showing gradual and obvious loss of nissl substance, black arrowhead with ne-neuronal swelling and edema, ns-neuronal shrinkage with condensation of nissl substance, i-irregular shaped degenerating neuron; **(H,J)** 24 and 72 h ZLHXTY-HD treatment, showing evident increase in nissl substance in neurons. 50 µm scale bar corresponds to 400 × magnification.

Microscopy revealed that the brain sections of mice from normal control group had no pathological changes following HE staining. Neuropil was intact with normal texture; and healthy, nucleated pyramidal neurons were clearly observed (Figure 3A). In 24 h ICH model group, subtle pathologic changes were observed in perihematomal region of brain. The neuropil was found to be less intact as compared to normal brain with signs of vacuolation, parenchymal loss, granulovacuolar neuronal degeneration, neuronal shrinkage, reactive gliosis including relatively excessive number of oligodendrocytes, astrocytes and microglia (Figure 3B) which became more obvious after 72 h (Figure 3D) with evident edematous changes. ZLHXTY-HD capsule treatment for 24 h maintained the normal neuropil architecture with less neuronal degeneration and less reactive gliosis (Figure 3C) observed. These protective changes were more obvious after 72 h of ZLHXTY capsule treatment (Figure 3E) showing that the histopathological damage of brain was reduced after treatment with ZLHXTY capsule.

Nissl staining was also used to identify the ICH induced neuronal injury since the loss of nissl substance indicate the damage to neurons. Figure 3F showed the presence of several blue coloured nissl bodies in pyramidal neurons in normal brain. After 24 h of ICH induction the number of nissl bodies were decreased (Figure 3G), that were further reduced after 72 h of ICH (Figure 3I). ZLHXTY-HD capsule treatment after 24 and 72 h as shown in Figures 3H,J, revealed the increased number of nissl stained neurons as compared to 24 and 72 h ICH model groups, indicating its neuroprotective effect.

### ZLHXTY Capsules Reduce the mRNA Levels of NFкB and Inflammatory Cytokines After ICH

**FIGURE 4:**
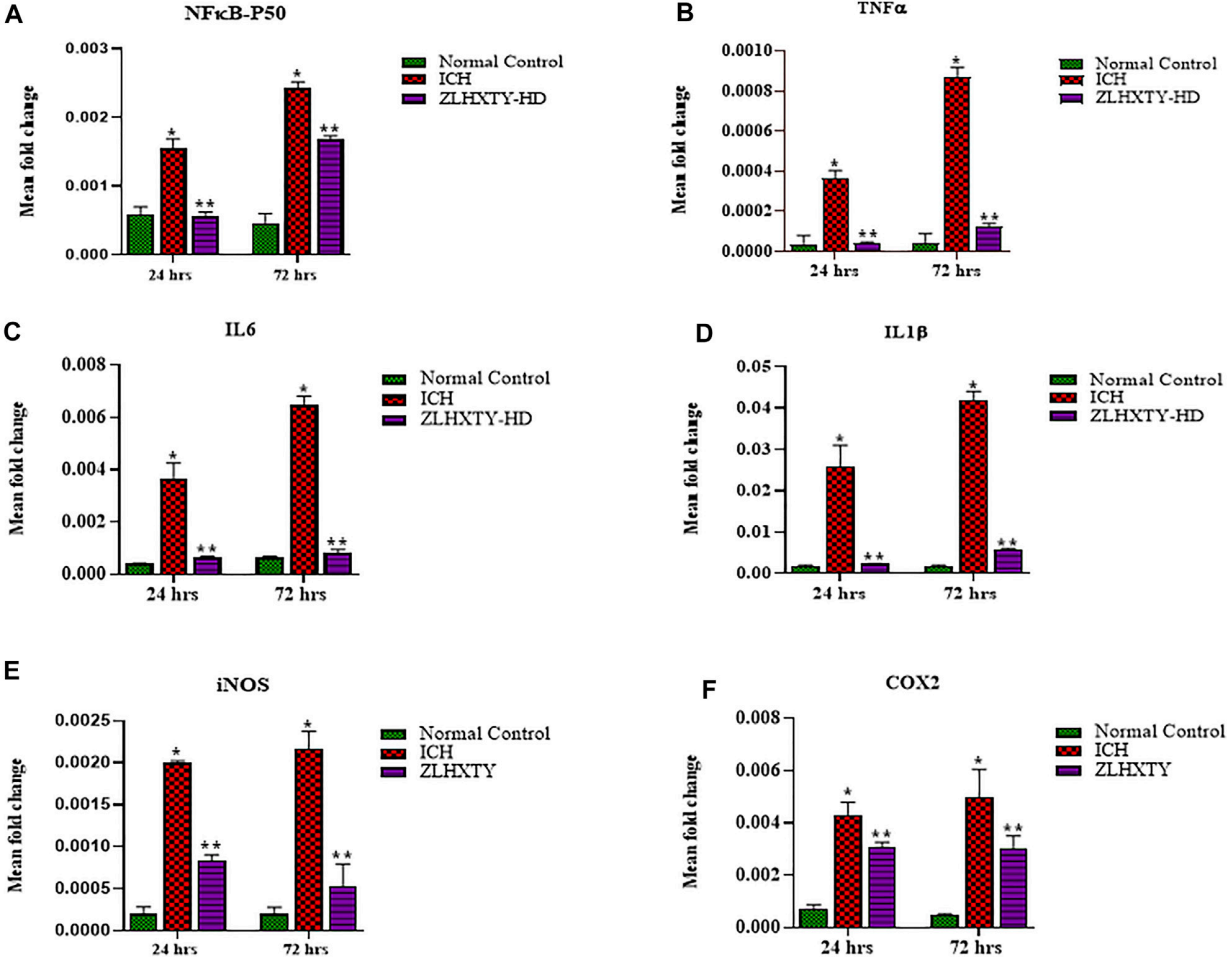
RT-qPCR revealed ZLHXTY treatment reduced mRNA expression of inflammatory cytokines. Graphs showing relative expression levels of mRNA **(A)** NFκB-P50, **(B)** TNFα, **(C)** IL6, **(D)** IL1β, **(E)** iNOS, and **(F)** COX2; in normal control, ICH and ZLHXTY-HD treatment groups after 24 and 72 h. The mRNA expression of inflammatory cytokines were reduced after ZLHXTY-HD capsule treatment for 24 h that becomes particularly significant after 72 h. Data represent the mean ± SD, *n* = 9, *p < 0.05 as compared to normal control, **p < 0.05 as compared to ICH group.

The mRNA expression levels of transcription factor, NFкB-P50, and its downstream target genes for inflammatory cytokines such as TNFα, IL6, IL1β, iNOS, and COX2 were assayed after 24 and 72 h of ZLHXTY treatment post-ICH. After 24 h of ICH, the values of all the measured inflammatory cytokines and NFкB were significantly higher than that in the normal control group (*p < 0.05), but significantly reduced with ZLHXTY-HD 24 h treatment (**p < 0.05 as compared to ICH group). After 72 h of ICH, the mRNA levels of NFкB and inflammatory cytokines were even higher than after 24 h of ICH, however, ZLHXTY-HD 72 h treatment significantly reduced the mRNA levels of NFкB and inflammatory cytokines (Figures 4A–F).

### ZLHXTY Capsules Negatively Regulate the Protein Expression of NFкB and Inflammatory Cytokines After ICH

**FIGURE 5:**
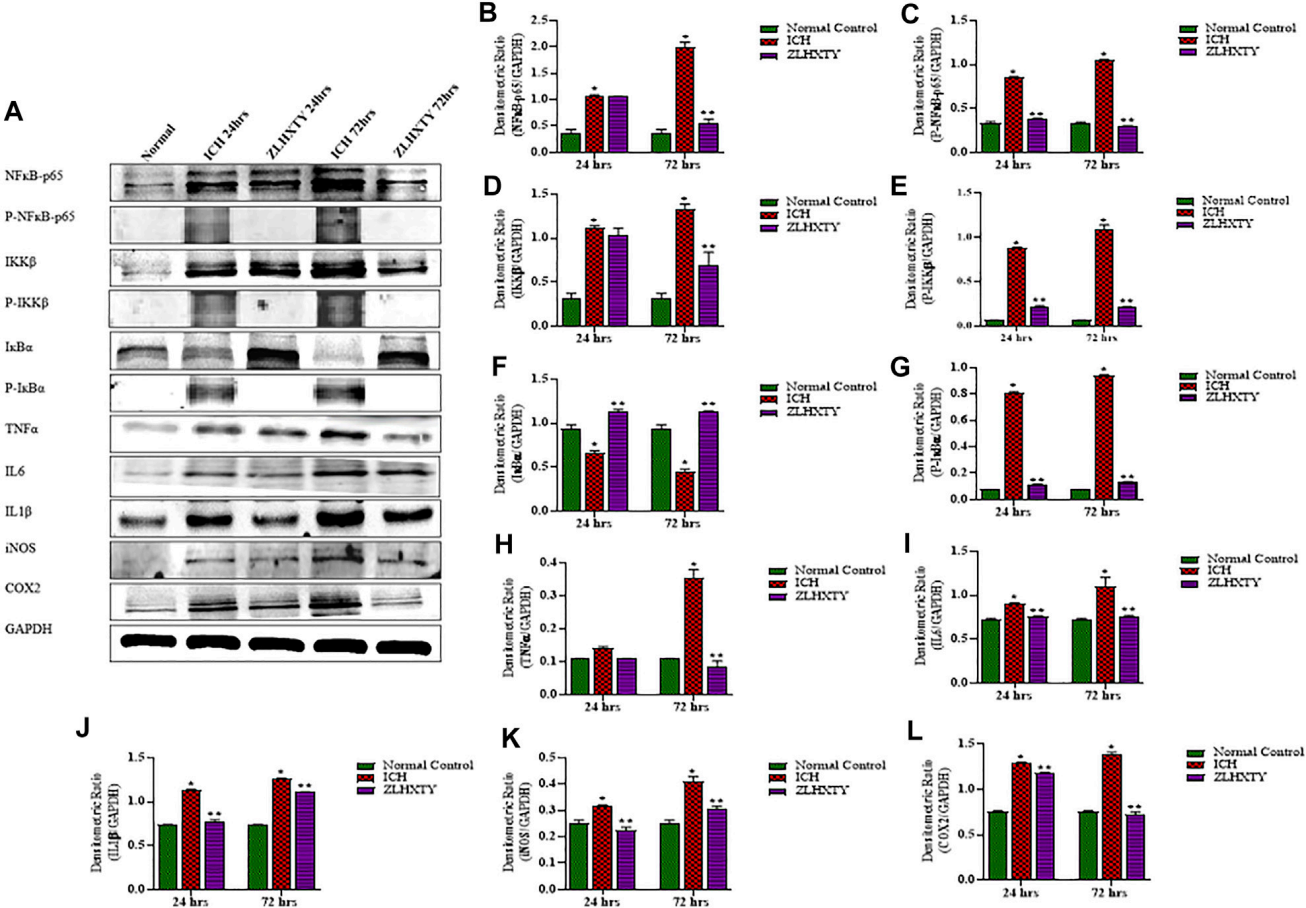
Western blotting demonstrated ZLHXTY-HD capsule downregulated the protein expression of NFκB signalling after ICH. **(A)** A representative immunoblot showing the effect of ZLHXTY-HD on protein expression of NFκB-p65, P-NFκB-p65, IKKβ, P-IKKβ, IкBα, P-IкBα, TNFα, IL6, IL1β, iNOS, COX2 and GAPDH; **(B)**, the quantitative densitometric ratio of NFκB-p65, **(C)** P-NFκB-p65, **(D)** IKKβ, (E) P-IKKβ, **(F)** IкBα, **(G)** P-IкBα, **(H)** TNFα, **(I)** IL6, **(J)** IL1β, **(K)** iNOS, and **(L)** COX2 relative to GAPDH. ZLHXTY-HD capsule treatment reduced the protein expression of NFκB-p65 and its target proteins after 24 and 72 h. Data represent the mean ± SD, *n* = 3, *p < 0.05 as compared to normal control, **p < 0.05 as compared to ICH group.

We further analysed the protein expressions of NFκB-p65, P-NFκB-p65, IKKβ, P-IKKβ, IкBα, P-IкBα, TNFα, IL1β, IL6, iNOS, and COX2 with western blotting after 24 and 72 h of ICH induction and ZLHXTY treatment. Figure 5A represent all the protein blotting results of our experiment. The total and phorphorylated protein levels of NFκB-p65 and IKKβ were significantly (*p < 0.05) upregulated after 24 h of ICH induction. Complementarily, the expression of total IKβα was reduced with increase in phosphorylated IKβα expression after 24 h of ICH. Concordantly, the protein levels of the inflammatory cytokines TNFα, IL6, IL1β, iNOS, and COX2 were also significantly (*p < 0.05) upregulated as compared to normal control group after 24 h of ICH. All these effects were significantly (*p < 0.05) augmented after 72 h of ICH. ZLHXTY-HD capsules induced significant (**p < 0.05) down-expression of NFκB-p65, P-NFκB-p65, IKKβ, P-IKKβ, TNFα, IL1β, IL6, iNOS and COX2. Whereas, the total protein expression of IKβα was upregulated and phosphorylated IKβα was downregulated after ZLHXTY treatment for 24 and 72 h (**p < 0.05) (Figures 5B–I).

### Immunofluorescence Co-Localization of TNFα and NFκB-p65 With NeuN and Iba1 Reveal Anti-Inflammatory Effect of ZLHXTY Capsules

**FIGURE 6:**
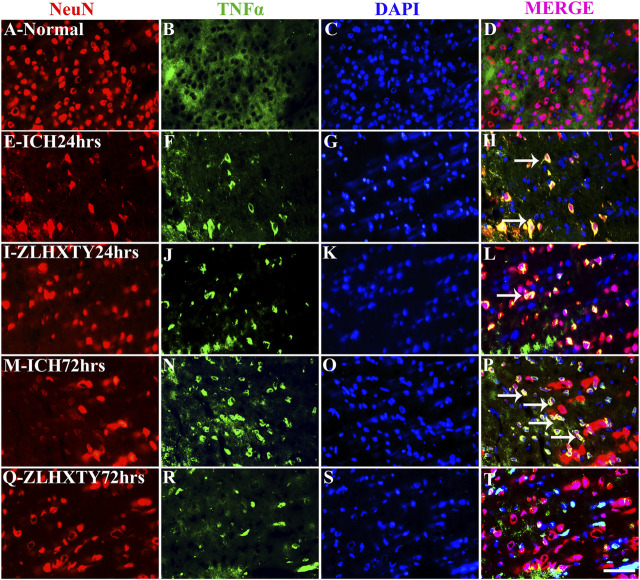
Double immunofluorescence of NeuN and TNFα. Brain sections were triple-stained with anti-NeuN (red), anti-TNFα (green) and DAPI (blue) to mark neurons, TNFα and nucleus. **(A–D)**, Normal brain section shows only NeuN staining. **(E–H)**, ICH 24 h group show decreased NeuN expression and increased neuronal TNFα expression, seen as yellow colour in merge. **(I–L)**, ZLHXTY 24 h group shows increased NeuN expression and reduction in TNFα expression. **(M–P)**, ICH 72 h show reduced NeuN expression and marked increase in neuronal TNFα expression (yellow in merge). **(Q–T)**, ZLHXTY 72 h group show reduced neuronal TNFα expression and increased NeuN. Respective change in color in merged figures D,H,L,P,T, corresponds to Red + Blue = Magenta; Red + Green = Yellow. 50 µm scale bar corresponds to 400 × magnification.

**FIGURE 7:**
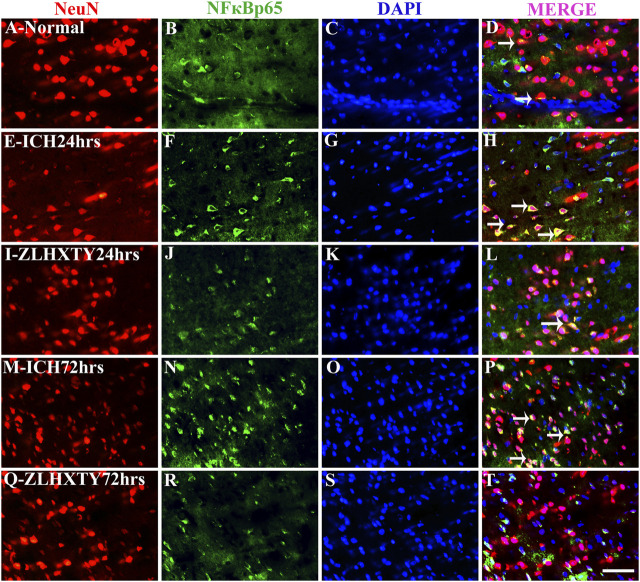
Double immunofluorescence of NeuN and NFκB-p65. Brain sections were triple-stained with anti-NeuN (red), anti-NFκB-p65 (green) and DAPI (blue) to mark neurons, NFκB-p65 and nucleus. **(A–D)**, Normal brain section shows very weak NFκB-p65 staining in some neurons co localized with NeuN. **(E–H)**, ICH 24 h group show increased neuronal NFκB-p65 expression with decrease in number of NeuN expressing neurons as compared to normal. **(I–L)**, ZLHXTY 24 h group shows increased NeuN number and reduction in NFκB-p65 expression. **(M–P)**, ICH 72 h show reduced NeuN expression in neurons co-expressing higher levels of NFκB-p65. **(Q–T)**, ZLHXTY 72 h group show obvious reduction in neuronal NFκB-p65 expression and increased NeuN number and expression. Respective change in color in merged figures D,H,L,P,T, corresponds to Red + Blue = Magenta; Red + Green = Yellow. 50 µm scale bar corresponds to 400 × magnification.

We further examined the expressions of TNFα and NFκB-p65 in neurons and microglia. In the normal control group, the uniformly distributed NeuN staining was observed throughout the brain tissue. The co-expression of TNFα and NeuN was not detectable in normal neurons whereas subtle expression of NFκB-p65 was noticeable in some neurons with very weak fluorescence (Figures 6A–D and Figures 7A–D). In the 24 h ICH group, the number and expression of NeuN was decreased compared to normal control, whereas, the expression of both the TNFα and NFκB-p65 was upregulated (Figures 6E–H and Figures 7E–H). This effect was further increased after 72 h of ICH (Figures 6M–P and Figures 7M–P). Interestingly, we found that positive expression of neuronal TNFα and NFκB-p65 was associated with very weak staining for NeuN. After 24 h of ZLHXTY-HD capsule treatment, the number of NeuN was increased and the fluorescence for TNFα and NFκB-p65 was reduced as compared to ICH 24 h group (Figures 6I–L and Figures 7I–L) which was markedly obvious after 72 h of ZLHXTY-HD capsule treatment (Figures 6Q–T and Figures 7Q–T).

**FIGURE 8:**
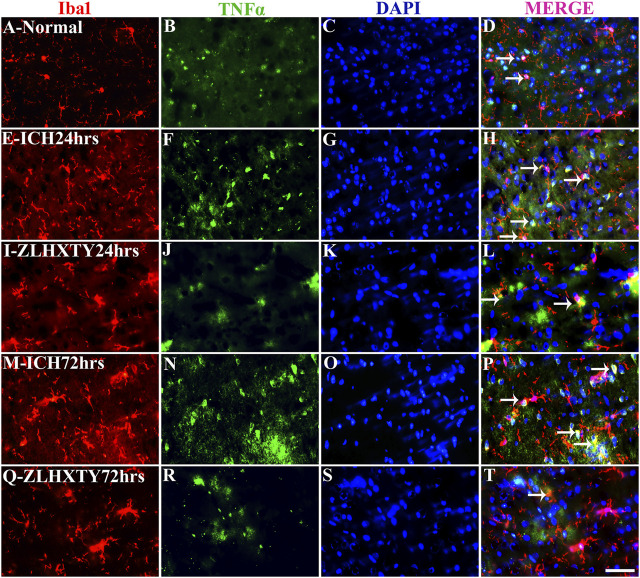
Double immunofluorescence of Iba1 and TNFα. Brain sections were triple-stained with anti-Iba1 (red), anti-TNFα (green) and DAPI (blue) to mark microglia, TNFα and nucleus. **(A–D)**, Normal brain section with few Iba1 positive cells and weak TNFα expressing cells. **(E–H)**, ICH 24 h group show little increase in number of Iba1 and marked increase in TNFα expression with colocalization signals as well. **(I–L)**, ZLHXTY 24 h group shows decreased Iba1 and TNFα expression as compared to ICH 24 h group. **(M–P)**, ICH 72 h show marked increase in both the signals of Iba1 and TNFα with co-localization. **(Q–T)**, ZLHXTY 72 h group show decreased expression signal of both Iba1 and TNFα and only few co-localization signals. Respective change in color in merged figures D,H,L,P,T, corresponds to Red + Blue = Magenta; Red + Green = Yellow. 50 µm scale bar corresponds to 400 × magnification.

**FIGURE 9:**
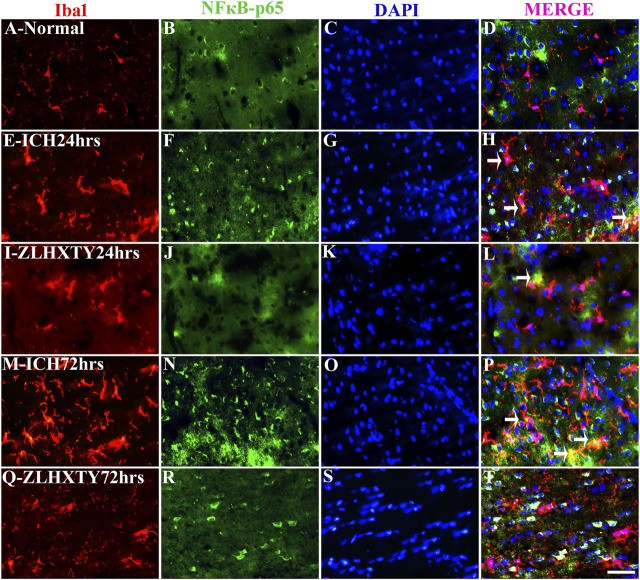
Double Immunofluorescence staining of Iba1 and NFκB-p65. Brain sections were triple-stained with anti-Iba1 (red), anti-NFκB-p65 (green) and DAPI (blue) to mark microglia, NFκB-p65 and nucleus. **(A–D)**, Normal brain section shows very few Iba1 stained glial cells and absence of NFκB-p65. **(E–H)**, ICH 24 h group show increased number and activation of Iba1 positive glial cells with a lot of projections and also increased NFκB-p65 expression but only few co-localization signals were found. **(I–L)**, ZLHXTY 24 h group shows decreased Iba1 activation and also negligible NFκB-p65 expression and very little co-localization signal was detected. **(M–P)**, ICH 72 h show immense activation of Iba1 stained glial cells and also NFκB-p65 expression and more co-localization signals as compared to ICH 24 h group. **(Q–T)**, ZLHXTY 72 h group show marked reduction in Iba1 and NFκB-p65 expression. Respective change in color in merged figures D,H,L,P,T, corresponds to Red + Blue = Magenta; Red + Green = Yellow. 50 µm scale bar corresponds to 400 × magnification.

The co-expression of TNFα and NFκB-p65 with Iba1 was also detected (Figures 8, 9). In normal brain sections, Iba1 expression was detected as few, small, compact soma bearing long, thin, ramified processes indicating inactivated form of microglia (Figures 8A–D and Figures 9A–D). After 24 h of ICH, the number of activated Iba1 positive cells were increased (Figures 8E–H and Figures 9E–H) with morphological changes i.e., cellular hypertrophy, membrane ruffling and retraction of processes. The co-expression of TNFα and NFκB-p65 with Iba1 was also increased as shown in Figures 8E–H and Figures 9E–H. These effects were further pronounced after 72 h of ICH (Figures 8M–P and Figures 9M–P). Conversely, after ZLHXTY treatment the number of Iba1 stained cells was controlled and also the expression of TNFα and NFκB-p65 was attenuated after 24 h (Figures 8I–L and Figures 9I–L) and more evident after 72 h (Figures 8Q–T and Figures 9Q–T).

## Discussion

TNFα-NFκB signalling pathway mediated inflammation and immune activation driven secondary brain damage is important pathological process to aggravate brain injury after ICH. In this study, we tested the hypothesis that ZLHXTY capsules protected against brain injury in the mouse model of blood induced ICH by improving the neurological scores and reducing brain inflammation when administered within 2 h of ICH induction. ZLHXTY capsules suppressed the inflammatory event following ICH by reducing the release of inflammatory cytokines and activation of leukocytes and microglia at the site of injury. It is assumed that the underlying mechanism of neuroprotection offered by ZLHXTY capsules may partly involve the down-regulation of the NFκB pathway. To the best of our knowledge, it is the first time to report that ZLHXTY capsules could protect against intracerebral hemorrhage at earlier stages *via* inhibiting NFκB canonical signalling (Supplementary Figure S1).

ZLHXTY capsule is a TCM formula composed of *Astragalus membranaceus* Fisch. ex Bunge (or *Astragalus mongholicus* Bunge), *Hirudo nipponica* Whitman, *Pheretima aspergillum* (E. Perrier), *Cinnamomum cassia* (L.) J. Presl (or *Neolitsea cassia* (L.) Kosterm.) and *Sargentodoxa cuneata* (Oliv.) Rehder and E.H. Wilson (or *Holboellia cuneata* Oliv.). For evaluation of quality standard of ZLHXTY capsule, due to the presence of multiple and complex chemical ingredients, we first performed a UPLC-HRMS quality control analysis. We identified the characteristic constituents present in the three herbs of ZLHXTY capsule and confirmed by using standard compounds. Since, *Hirudo nipponica* Whitman (Leech), and *Pheretima aspergillum* (E. Perrier) (earthworm) are animals, they mainly consists of proteins, peptides, amino acids, fatty acids, phospholipids, mineral substances, nucleosides and other compounds, therefore, out of scope in the currently used chromatographic conditions. Besides various therapeutic effects exerted *via* multiple signalling pathways, all these five Chinese medicines posses one common anti-inflammatory property through inactivation of NFκB signalling pathway (Zhao et al., 2015; Huang et al., 2016; Li et al., 2016; Lou et al., 2019; Yao et al., 2020).

Previous research suggests that neuronal activation of NFкB have multiple consequences on both the molecular level and behavioural outcomes. The regulation of cognitive behaviors in mice, including learning and memory involves mainly the role of NFкB family members, p50, c-Rel, and p65/RelA, as well as IKK (Dresselhaus and Meffert, 2019). In our study, we used three different sensorimotor neuro-behavioral tests to examine ICH-induced brain injury in the mice. All these tests were well suited to models of unilateral brain injury as well as for examining recovery of function after ICH (Hua et al., 2002). Our study have demonstrated that ZLHXTY capsules improved neuro-behavioural outcomes in a dose dependent manner. The ZLHXTY-HD capsules significantly reduced the neurological deficit scores, improved overall performance at balance beam and produced better control over percentage of right turns in a corner turn test in ICH mouse model. Therefore, we selected the highest dose for all the subsequent experiments. These preliminary tests provide better insight of the brain damage after ICH that mainly targets basal ganglia. Neurological scoring examine sensorimotor function of animals (Clark et al., 1997; Ruan and Yao, 2020). Balance beam test is a highly sensitive method to assess motor co-ordination after ICH (Feeney et al., 1982; Liu et al., 2020). Corner turn test is also specific to indicate the unilateral brain injury and resulting hemiplegia after ICH (Krafft at el., 2014; Schaar et al., 2010).

Primary ICH injury occurs soon after the onset of hemorrhage with the formation of hematoma, mass effect, increased intracranial pressure and mechanical disruption of adjacent tissues. The presence of intraparenchymal blood leads to secondary damage which involves activation of cytotoxic, excitotoxic, oxidative and inflammatory pathways causing neuronal apoptosis, inflammation, and cerebral edema (Felberg et al., 2002; Keep et al., 2012). This initial cascade of neuronal death, localized immune activation and inflammation occurs soon after the onset of ICH (0–6 h) and continues to propagate to perihematomal regions for further brain damage in between 12 and 72 h, contributing to the secondary brain injury (Xue and Del Bigio, 2000; Felberg et al., 2002; Keep et al., 2012). Therefore, it is desirable that neuroprotective anti-inflammatory interventions should be commenced at earlier stages after the onset of ICH that may provide substantial benefit to ICH patients (Askenase and Sansing, 2016). In our study, we found that ZLHXTY capsules demonstrated effective anti-inflammatory and neuroprotective effects when administered within 2 h after the induction of ICH.

Inflammation is a primary immune response to infection or tissue injury and a protective adaptation for tissue homeostasis that resolves over a period of time, otherwise may lead to acute or chronic inflammatory diseases (Ahmed, 2011). NFκB is a central mediator of inflammation and functions in both innate and adaptive immunity, cellular differentiation, proliferation, and survival in multicellular organisms (Mitchell et al., 2016). After ICH, NFκB is activated within minutes and lasts for at least 1 week. Several studies indicate that RBCs and plasma play role in activation of NFκB via signalling pathways involving free radicals, cytokines and glutamate receptors (Fang et al., 2013). The activity of NFκB positively correlates to the perihematomal neuronal cell death after ICH in both preclinical and clinical observtaion (Wang et al., 2011a; Fang et al., 2013). Concordantly, we also observed increased neuronal damage in both HE and Nissl stained brain sections from ICH group. While treatment with ZLHXTY capsules protected from neuronal death.

NFκB is a key transcription factor for induction of a inflammatory genes encoding TNFα, IL6, IL1β, IL-12p40 and COX2 in various pathological conditions, including ICH (Liu et al., 2017). The initial 72 h after ICH are critical for inflammatory brain injury, indicating higher expression levels of pro-inflammatory cytokines (Zheng et al., 2015; Askenase and Sansing, 2016). In agreement to this observation, we have also found upregulated mRNA and protein expression of NFкB and inflammatory cytokines TNFα, IL6, IL1β, iNOS, COX2, upon ICH induction that peaked at 72 h, but inhibited by ZLHXTY capsules treatment, indicating its anti-inflammatory effect.

NFκB signalling is regulated by two pathways, the canonical, NFκB essential modulator NEMO dependent pathway and the non-canonical, NEMO independent pathway (Mitchell et al., 2016). The canonical pathway is triggered by pro-inflammatory cytokines such as TNFα and IL1 (Karin and Ben-Neriah, 2000) derived from astrocytes, neurons and majorly microglia/macrophages (Wang et al., 2011b). Whereas, the alternative pathway is activated by lymphotoxin *β* (LTβ), CD40 ligand, B cell activating factor (BAFF), and receptor activator of NFκB ligand (RANKL), but not TNFα (Dejardin et al., 2002; Lawrence, 2009). Therefore, in our study we have focused on the role of canonical NFκB signalling in ICH. It is suggested that after ICH, the monocyte derived cytokines, TNFα and IL1β, recruit highly activated blood-derived macrophages and neutrophils to the perihematomal region, that further release inflammatory factors and aggravate inflammatory brain damage. These inflammatory signals including thrombin, heme, and high mobility group box 1 (HMGB1) bind specific cell surface receptors on myeloid cells, resulting in NFκB activation (Askenase and Sansing, 2016). The distinguishing feature of canonical regulation of NFκB pathway involves the activation of RelA(p65) or cRel containing complexes, and require IKKꝨand IKKβ subunits of IKK signalsome assembly for phosphorylation and degradation of IкBα, the inhibitor of NFκB. In contrast, the alternative pathway involves activation of RelB/p52 complexes, and requires only IKKα subunit of signalsome for phosphorylation and processing of p100/p52, and does not involve IκBα degradation (Lawrence, 2009). In normal states, the inactive form of NFκB-p65 (p50/p65) is maintained by its continuous phosphorylation, ubiquitination and proteasomal degradation in the cytosol through interaction with IкB inhibitor proteins, required to prevent unnecessary immune activation and maintaining homeostasis (Lawrence, 2009; Oeckinghaus and Ghosh, 2009). However, upon stimulation to various inflammatory cytokines, a signalling cascade is initiated that leads to phosphorylation and activation of IKKβ (a subunit of the inhibitor of IκB kinase, IKK, complex). Activated IKKβ leads to phosphorylation of the IκB inhibitory protein IκBα, for subsequent ubiquitination and proteasomal degradation, and consequent release of NFκB (p65/p50) to the nucleus. The activated NFκB (p65/p50) binds to its recognition sites on DNA sequences to induce target gene expression encoding for proinflammatory cytokines and cell survival (Ghosh and Karin, 2002; Lawrence, 2009; Oeckinghaus and Ghosh, 2009).

In the canonical NFкB pathway, the critical member of signalsome, IKKβ, is both necessary and sufficient to phosphorylate IкBα and IкBβ regulated by IKKꝨ. Thus, several *in-vitro* and *in-vivo* IKKβ gene knock out studies exhibit defective TNFα or IL1β signalling to NFкB (Ghosh and Karin, 2002; Oeckinghaus and Ghosh, 2009; Brasier, 2010; Frakes et al., 2014; Dresselhaus and Meffert, 2019). IKKβ is the major effector IкBα kinase, which serves as an essential adapter organizing the activated, high molecular weight complex, IKK signalsome assembly that binds ubiquitylated signalling adapters, and recruits the IкBα inhibitor into the activated IKK complex (Brasier, 2010). Although both of the IкBα and IкBβ are believed to inhibit c-Rel and p65 containing complexes, IкBα is the best-studied member of the IкB family displaying all the characteristics of an NFкB inhibitor and have shown a higher affinity for p65:p50 complexes (Malek et al., 2003; Oeckinghaus and Ghosh, 2009).

Therefore, to study canonical signalling, we selected these two critical molecules IKKβ, subunit of signalsome and IкBα inhibitor. Through our western blot results, it is evident that upon ICH, the protein expression level of total and phosphorylated NFкB and IKKβ are upregulated with inhibition of total IкBα inhibitor that undergo increased phosphorylation. Conversely, treatment with ZLHXTY reverse these effects.

Neurons are known to express NFκB under basal conditions to maintain health, synapse growth and plasticity-related functions, while, under disease conditions, NFκB is upregulated. In glial cells, NFκB is reported to have little basal activity but chronic or excessive glial activation of NFκB has shown to be neurotoxic. Microglia are the innate resident phagocytes of the CNS that upon inflammatory stimulus becomes activated and express NFκB with subsequent expression of TNFα and IL1β (Askenase and Sansing, 2016; Dresselhaus and Meffert, 2019). Our immunofluorescence results also showed increased neuronal NFκB and TNFα expression after 24 and 72 h of ICH. Further, the microglial activation was also observed after ICH through Iba1 staining, however, ZLHXTY attenuated that effect.

After initial hemorrhage, the process of inflammation continues to develop over many days, within the CNS, thus, inflammation may represent an ideal target for treatment of the disease. In this study, we have focused on inflammatory pathway regulated by NFкB canonical signalling, however, further research is required to identify other mechanisms that promote inflammatory pathways. Basal regulation of neuronal activity by NFкB signalling is also less studied area and requires to be unveiled. Moreover, we studied inflammation only for 72 h post-ICH that we observed to be the peak time of brain injury. However, further elaborate work must be required to identify and explain the role of NFкB signalling and its regulatory effect on neurons and microglia upon overall brain function over extended period of time after ICH.

## Conclusion

To conclude, ZLHXTY capsules improve neurological outcome, alleviate brain damage by inhibiting the inflammatory response and that may occur by negative regulation of NFкB signalling in a classical manner. Our findings suggest that early administration of ZLHXTY capsule provide neuroprotection after ICH and can serve as an effective treatment option.

## Data Availability

The original contributions presented in the study are included in the article/Supplementary Material, further inquiries can be directed to the corresponding authors.

## Supplementary Material

Supplementary Figure S1 Graphical Abstract: Pictorial representation of the anti-inflammatory mechanism of Zhilong Huoxue Tongyu capsule for the treatment of intracerebral hemorrhage.
