# Supplementary figures and images for: Zhilong Huoxue Tongyu Capsules Ameliorate Early Brain Inflammatory Injury Induced by Intracerebral Hemorrhage via Inhibition of Canonical NFкβ Signalling Pathway
Source: Front Pharmacol. 2022 Mar 31;13:850060. doi: 10.3389/fphar.2022.850060 (PMC9008889; doi:10.3389/fphar.2022.850060)

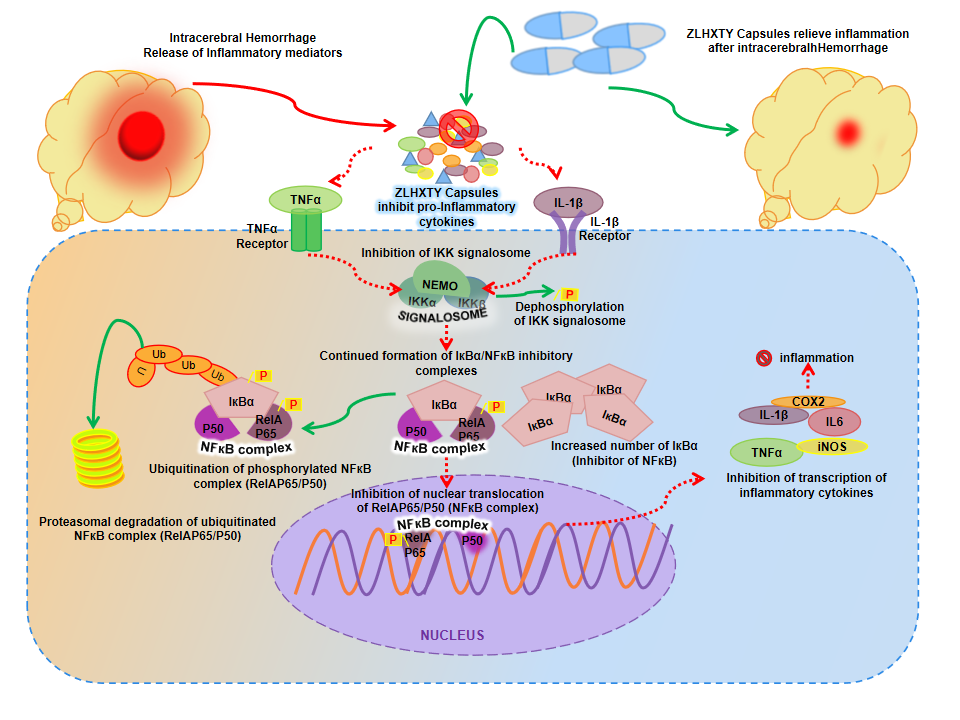

Supplement: Supplementary file 1 [file Image1.TIF]
